# Supplementary figures and images for: Characterization of human exposure to Anopheles and Aedes bites using antibody-based biomarkers in rural zone of Cameroon
Source: PLoS One. 2024 Dec 5;19(12):e0314709. doi: 10.1371/journal.pone.0314709 (PMC11620597; doi:10.1371/journal.pone.0314709)

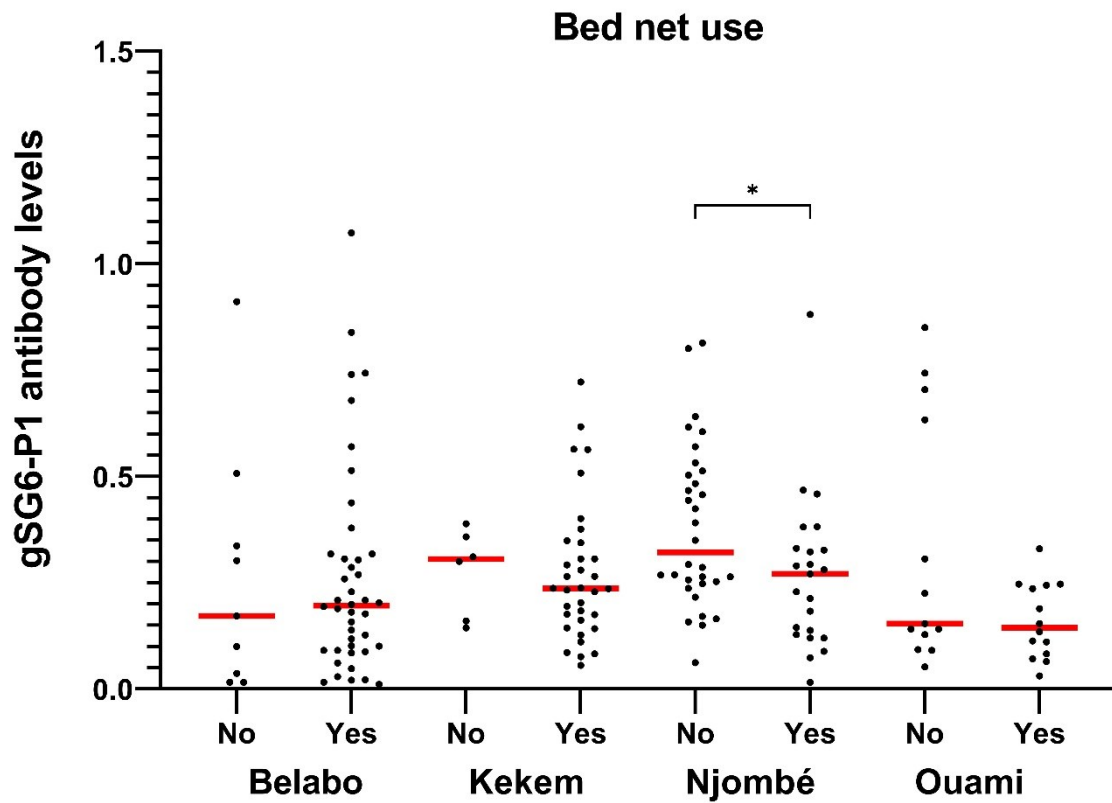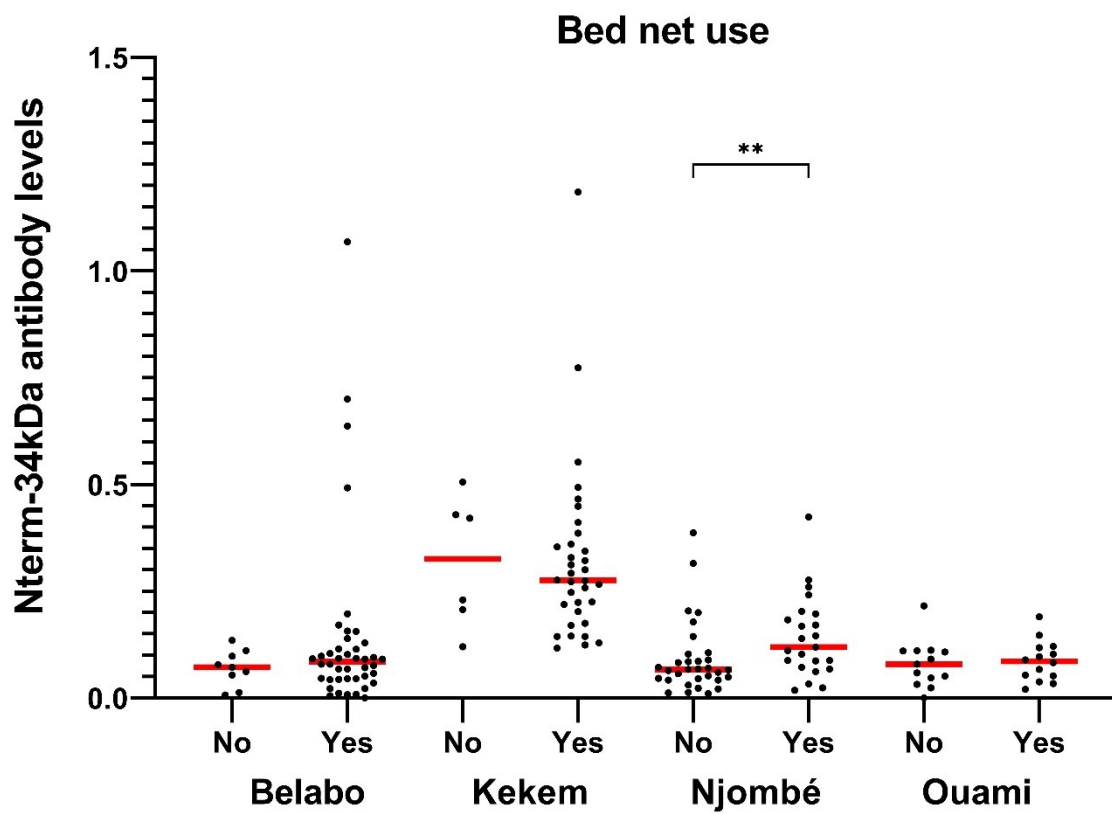

Supplement: S1 File — Red bars represent median values in each village. (PDF) [file pone.0314709.s001.pdf]
